# Self-Managing Postoperative Pain with the Use of a Novel, Interactive Device: A Proof of Concept Study

**DOI:** 10.1155/2016/9704185

**Published:** 2016-04-30

**Authors:** Luke Mordecai, Frances H. L. Leung, Clarissa Y. M. Carvalho, Danielle Reddi, Martin Lees, Stephen Cone, Zoe Fox, Amanda C. de C. Williams, Brigitta Brandner

**Affiliations:** ^1^University College London Hospital, London NW1 2BU, UK; ^2^UCL and the Education Unit, Biomedical Research Centre, UCL Institute of Neurology, London WC1E 6BT, UK; ^3^Research Department of Clinical, Educational & Health Psychology, University College London, London WC1E 6BT, UK

## Abstract

*Background*. Pain is commonly experienced following surgical procedures. Suboptimal management is multifactorial.* Objectives*. The primary objective was to assess whether patients used a device (Navimed) to self-report pain over and above a normal baseline of observations. Secondary outcome measures included comparison of pain scores and patient use of and feedback on the device.* Methods*. In a prospective randomized controlled trial, elective gynaecological surgery patients received standard postoperative pain care or standard care plus the Navimed, which allowed them to self-report pain and offered interactive self-help options.* Results*. 52 female patients, 26 in each of device and standard groups, did not differ in the frequency of nurse-documented pain scores or mean pain scores provided to nurses. The device group additionally reported pain on the device (means 18.50 versus 11.90 pain ratings per day, *t*(32) = 2.75, *p* < 0.001) that was significantly worse than reported to nurses but retrospectively rated significantly less anxiety. 80% of patients found the device useful.* Discussion and Conclusion*. This study demonstrates that patients used the Navimed to report pain and to help manage it. Further work is required to investigate the difference in pain scores reported and to develop more sophisticated software.

## 1. Introduction

Moderate to severe acute pain is commonly experienced in medical and surgical patients. Pain management in inpatient settings is hampered by shortcomings in pain assessment and delays in pain treatment [1]. Devices such as patient controlled analgesia (PCA) have improved satisfaction [2] due to the immediacy of effective pain relief as well as giving the patient a sense of empowerment [3, 4]. Negative pain experiences are often due to pain not being acknowledged, delay in pain relief, and patients being labelled as “difficult” [5]. A patient's perceived lack of control regarding both the events and the environment in hospital, in addition to poor communication from healthcare professionals, may exacerbate a negative experience [6].

Psychological factors also form a key part of pain experience [7]. They exert control over sensory input and determine patients' responses and are thus very important in the management of pain. There are various routes for intervention, from changing expectations to supporting coping strategies.

Providing patients with procedural information during an intervention reduces pain scores, total analgesic use, and length of hospital stay [8]. The most effective information includes a detailed sensory description of what the patient is likely to feel, decreasing pain scores and distress [9].

There is also evidence that relaxation techniques, which can be delivered by audio or written instruction, as well as imagery and music, have an adjuvant role in alleviating pain. Relaxation focuses on reducing muscle tension and calm breathing techniques; music may induce these but is also distracting and may reduce subjective awareness and distress [7]. There are several systematic reviews that support the use of these methods [10, 11].

Attention diversion techniques can also modulate pain experience. Audio or visual stimuli may be used to achieve this and can also be used concomitantly alongside relaxation techniques already discussed [12]. A Cochrane review found that listening to music could reduce postoperative pain intensity and opioid requirement [13] as well as decrease anxiety [14].

The Navimed introduces nonpharmacological management of pain into the clinical environment, under the patient's control, with the intention of providing the means to self-report pain whenever the patient wishes; to provide procedural information available at any time; and to provide relaxing and attention diverting resources to help manage postoperative pain. The choice of attention diverting resources was based on discussion with pain management clinicians.

Existing applications rely heavily on providing information or on single modality strategies, and none have been formally evaluated [15]. A recent review article evaluated 220 applications available to download for chronic pain in the US. Half pertained to chronic nonspecific pain and a further quarter to neck and back pain. Over 90% of the applications focused on either information regarding the condition or self-management and most appeared to have no input from healthcare professionals nor include any evidence based on pain management features [16]. The Navimed is the first to combine multiple approaches within the same device and provide an interactive experience as well as being designed by a specialist multidisciplinary pain team.

## 2. Methods

A prospective, unblinded, randomised controlled trial was carried out at a large London teaching hospital. Patients were selected, as per the inclusion criteria, and consented at routine surgical preassessment visits at the hospital.

Patients were randomised by a researcher not involved in the allocation process, by sealed envelope, to receive standard postoperative care (Group S) or standard postoperative care plus a handheld device Navimed (Group D). The patients in Group D were instructed preoperatively how to use the Navimed and familiarised themselves with a training version in the presence of a researcher. They were issued with their own device in recovery when fully awake postoperatively and reminded again how to use it. The trial started once the participant was transferred to the surgical ward.

Both Group S and Group D had their pain scores recorded by nursing staff at 4 hourly intervals in accordance with hospital guidelines using a 0–4 scale with the verbal labels none, mild, moderate, severe, and very severe. Group D could also report pain scores on the device as frequently or infrequently as they desired on a 0–4 scale from least (0) to most (4) as well as access preloaded media content designed specifically for this trial. In order to access the media content, participants had first to enter a pain score. Nurses did not see the scores entered on the Navimed.

The study lasted for 48 hours, after which time each participant completed a series of questions assessing her satisfaction with pain control and evaluating the Navimed. In the event, a patient was discharged before 48 hours; this assessment was performed just before the patient left the hospital. Participants were free to withdraw from the study at any time. Reasons for withdrawal or dropout from the study were recorded.

### 2.1. Participants

Patients were initially approached at routine surgical preassessment appointments at the hospital, which they attended at least two weeks prior to booked gynaecological procedures such as a laparoscopic hysterectomy. Those meeting inclusion criteria were given an information leaflet regarding the study and the opportunity to speak with one of the doctors or nurses on the research team. A member of the research team obtained informed, written consent from patients prior to anaesthesia on the day of surgery. The study was performed over the period beginning from 13th November 2013 to 25th March 2014 inclusive.

The inclusion criteria include

- being female,
- age 18–90,
- elected patient,
- gynaecological surgical patients at UCLH,
- routine ward care postoperatively anticipated for at least 48 hours.

The exclusion criteria include

- chronic pain diagnosis,
- intravenous drug users and other substance abusers,
- being unable to read or understand English (since device is programmed in English),
- being unable to give consent or lacking capacity.

The main research questions were (1) whether patients used the Navimed device to record their pain in addition to regular pain monitoring by nurses and, (2) if they did, whether the pain scores on the Navimed differed significantly from those they reported to nurses. We were also interested in whether patients used the multimedia programmes loaded onto the device.

To calculate power, we used pilot data on 19 subjects of a previous study in which the mean frequency of nurse pain assessments in the 48 hours following surgery was 9.70 (SD 2.40), somewhat less than that required by hospital guidelines (12 4-hourly observations in the 48 hours following surgery). The minimum clinically important difference we wished to detect was a 50% increase in the frequency of observations to a mean of 14.60 using the Navimed. For this calculation, we assumed a standard deviation of 5 in both arms, a 5% noncompliance rate, and 1% dropout in the 48 hours following surgery. For 90% power and a two-tailed significance level of 5%, sample size required was 52.

### 2.2. Interventions

Patients randomised to use the Navimed were trained prior to their surgical procedure. The device has a touch screen and operates much like smartphones and tablets. Patients recorded a pain score, at rest and on movement, whenever they chose. They would do this by selecting a tab to rate their pain on the Navimed device and then by selecting a number between 0 and 4. After recording a pain score on the device, patients were presented with a number of options relating to further activities. These were

1. advice: about the nature of postoperative pain; about medication side effects; about how to use a PCA effectively,
2. comedy: four audio tracks about London landmarks,
3. film or TV: short nature films and cartoons,
4. games: a jigsaw,
5. guided relaxation: two spoken tracks (one progressive relaxation and one mindful breathing), designed for postoperative patients in the trial, and two music tracks with nature pictures,
6. guided stretch: four videos of stretches done sitting on the bed, with spoken instructions, produced by a pain physiotherapist for the trial,
7. information: about the acute pain team in the hospital, pain control options, and controlling side effects of drugs,
8. music: Latin, blues, African, Brazilian, guitar, orchestra, and rock,
9. talking books: four nonfiction stories.

These options were selected from discussions within the acute pain team of the hospital on the basis of expert experience and best evidence as discussed in the introduction. The use of the Navimed device was automatically recorded on the device for each patient and subsequently downloaded and analysed by the research team.

Patients in both groups had the option to call for the nurse using the usual bedside call button at any time.

### 2.3. Outcome Measures

The primary study objective was to assess whether patients used a handheld device to self-report pain over and above a normal baseline of nurse observations collected during usual clinical care.

Secondary objectives were (1) to compare the device reported pain scores with the nurse-recorded pain scores (both 0–4), (2) to compare the pain scores between patients using the handheld device alongside standard care and those receiving standard care alone, and (3) to compare the qualitative and quantitative assessment of usefulness of the Navimed from the patients' perspective.

All participants were asked 20 questions about their postoperative experience: 17 using numerical rating scales and three closed questions regarding recollections: see Table 6 and Supplementary Material available online at http://dx.doi.org/10.1155/2016/9704185 for questions. They were also given the opportunity to write free text regarding their experience of postoperative pain management. Participants that used the Navimed were then asked further three closed questions and 7 numerical rating scales specifically about the device, with another free text space to feed back about potential improvements. All questions may be found in Supplementary Material.

## 3. Results

Three people eligible to enter the trial declined to do so. The dropout rate after 48 hours, shown in Table 1, was due to three early discharges and two software malfunctions in the Navimed. There were no differences between groups in patient ages or type of surgery, as shown in Table 2.

### 3.1. Pain Scores

To assess whether patients used the handheld device to record pain scores in addition to standard nurse-collected pain observations, the total number of pain scores in each group was compared. The total number of pain scores was significantly higher in the Navimed than in the standard group (means 34.19 (SD 18.91) and 23.27 (SD 7.23), resp., *t*(50) = 2.75, *p* = 0.0080). This difference was accounted for by use of the Navimed since the number of nurse observations did not differ across the two groups (mean 22.62 (SD 8.20) and 23.27 (SD 7.23) (*t*(50) = 0.305, *p* > 0.05)); see Tables 3 and 4.

Given the use of the Navimed, we tested for difference within Group D in mean pain scores reported on the Navimed and to nurses. Significantly greater pain was reported on the Navimed than to a nurse: means 1.02 (SD 0.87) and 0.28 (SD 0.29), respectively,* t*-test (*t*(50) = 4.07, *p* < 0.0010). Comparison between Groups S and D of the pain scores reported to nurses showed no significant difference over the 48-hour period (*t*-test, *t*(47) = 0.857, *p* > 0.050).

The retrospectively rated worst pain reported was 5.80/10 (SD 2.70) in Group D with only one of the participants complaining of 10/10 severity and 7.20/10 (SD 2.60) in Group S with seven recalling 10/10 pain, but this difference was not statistically significant (*t*(50) = 1.94, *p* > 0.05). Both groups estimated spending about 30% of the time in severe pain. Least reported pain showed a floor effect, with a median score of 1.50/10 in both groups.

### 3.2. Mood and Interference by Pain

Table 6 shows the scores of pain and mood (see Supplementary Material) completed by all patients after they finished the trial.

Pain interference with activities in bed, such as sitting up (Group S mean 5.81, SD 3.29, and Group D mean 5.08, SD 3.01; *t*(50) = 0.83, *p* > 0.05), with breathing deeply/coughing (Group S mean 4.77, SD 3.42, and Group D mean 4.00, SD 2.99; *t*(50) = 0.86, *p* > 0.05), with sleep (Group S mean 3.85, SD 2.92, and Group D mean 3.85, SD 2.95; *t*(50) = 0.00, *p* > 0.05), and with getting out of bed (Group S mean 4.75, SD 2.91, and Group D mean 3.72, SD 2.72; *t*(47) = 1.28, *p* > 0.05) did not differ between groups (see Table 6). By contrast, anxiety caused by pain was significantly lower in Group D, mean 2.90 (SD 2.90), compared to Group S, mean 4.70, SD 3.40: *t*(50) = 2.16, *p* = 0.036. For distress, the scores were Group D mean 3.30 (SD 3.20) and Group S mean 5.00 (SD 3.30), which was not statistically significant: *t*(50) = 1.82, *p* = 0.070.

### 3.3. Pain Control Overall

Overall relief, rated as a percentage, from analgesics was similar across groups (Group S mean 73.85, SD 25.93, and Group D mean 77.31, SD 21.83; *t*(50) = 0.52, *p* > 0.05) as were the number of patients who would have liked more pain relief (Group D, 7; Group S, 5) and the number who reported receiving information about options for pain treatment (Group D, 18; Group S, 19). Nor were there differences in the ratings of participation in decisions about pain treatment, satisfaction with pain treatment since surgery, with a mean just above 8/10 in both groups, and overall satisfaction with experience of pain control. For data, see Table 6.

There were no notable differences in extent of nausea (Group S mean 5.15, SD 4.05, and Group D mean 3.73, SD 3.47; *t*(50) = 1.36, *p* > 0.05), drowsiness (Group S mean 6.04, SD 3.41, and Group D mean 4.65, SD 3.26; *t*(50) = 1.50, *p* > 0.05), or itchiness (Group S mean 2.04, SD 2.95, and Group D mean 1.77, SD 2.61; *t*(50) = 0.35, *p* > 0.05), although patients with the device did retrospectively report significantly less dizziness (Group S mean 4.42, SD 3.71, and Group D mean 2.54: *t*(50) = 2.04, *p* = 0.047).

### 3.4. Use of the Navimed

After using the Navimed, 21/26 (81%) were satisfied with it and 20/26 (77%) found it very easy or easy to use. Patients used the full range of options available on the device; the three most commonly accessed functions were information, guided relaxation, and games (see Table 5).

## 4. Discussion

The results from this pilot study demonstrate that patients made full use of the Navimed device and the majority found it helpful and easy to operate. This answers the primary objective of the study and confirms that further research is warranted regarding development of this concept. It is also reassuring to see that the presence of the Navimed device did not detract from normal level of attention from healthcare staff, with no significant difference between groups in the number of nursing pain observations.

The significantly higher mean pain score recorded on the Navimed compared to scores reported to nurses was unexpected but is likely to reflect the fact that the nurse-recorded pain score involves a social interaction with both sides potentially able to influence the outcome [17]; for instance, a patient may perceive pressure to represent herself as stoical or coping well or may not want to make demands of staff [17]. It is also worth noting that while both the nurses and Navimed used a 0–4 scale to rate pain, there was a minor difference in presentation of the scales, an inadvertent discrepancy that arose through lack of coordination between the programming and clinical teams.

The secondary outcomes were exploratory and we did not power the trial to investigate them but to guide subsequent work. Most notable among these secondary outcomes is the apparent reduction in patient anxiety experienced in the device group, possibly associated with free access to information about pain, one of the most used functions, and with a sense of empowerment conferred by using self-initiated nonpharmacological methods to reduce pain distress. These ratings are subject to retrospective bias and imperfect recall compounded by a general anaesthetic and strong intravenous analgesics, but those considerations affect scores from both groups. Additionally, the retrospective report of pain is affected by a “peak end bias” whereby average pain rating over a given period is biased by the worst pain recalled and the pain at the end of the experience [18]. These limitations suggest caution in interpreting differences in pain scores using this methodology between the two groups.

In a seminal publication, the Harvard economist Michael Porter describes better value in healthcare as improved outcomes with reduced costs [19], and patient engagement is increasingly being recognised as one important conduit to deliver this [20]. This study strongly suggests that postoperative patients want to engage and be involved in their care. The Navimed is defined by this patient engagement and empowerment and excitingly could prove to be a disruptive innovation in the entire patient hospital experience.

In order to raise awareness and improve management, there have been recent suggestions that pain should be treated as “the fifth vital sign” [21] and evidence to suggest treating it in this manner improves outcomes [22]. The mantra of assess, treat, and reevaluate is a sensible and universally adopted mechanism, but robust evidence is lacking on how often pain observations should be taken, especially in the context of hard-pressed clinical staff on busy surgical wards. The Navimed can bypass this issue as it offers the potential for continuous assessment, which with an appropriate network could be monitored in real time by the hospital acute pain team, allowing a rapid and specialist response to patients with poorly controlled pain. Further development, including integration in the hospital information system, is underway.

## Supplementary Material

The supplementary material provided is the post trial questionnaire given to all participants at the conclusion of their involvement. It asks questions regarding the retrospective experience of pain as well other symptoms and emotions using both closed questions and numerical rating scales. Those participants that used the Navimed were then asked a further series of questions specifically regarding their usage of, and satisfaction with the device. The results from this questionnaire are included within the main text of the article.

## Figures and Tables

**Table 1:** The number of patients with data collected during each day of the trial.

Time-point	Group S	Group D
Baseline (*n*)	26	26
24 hours	26 (100%)	26 (100%)
48 hours	25 (96.2%)	22 (84.6%)

**Table 2:** The patient population and descriptions of surgeries performed. There was no statistically significant difference between populations.

	Group S *n* = 26	Group D *n* = 26
Age (yrs); mean (SD)	50.4 (15.9)	49.0 (13.4)
Surgical procedure: *n* (%)		
Colposuspension	2 (7.7%)	2 (7.7%)
Adhesiolysis	0 (0.0%)	3 (11.5%)
TAH/hysterectomy	12 (46.2%)	11 (42.3%)
Myomectomy	3 (11.5%)	3 (11.5%)
Endometriosis	4 (15.4%)	4 (15.4%)
Other procedures	5 (19.2%)	3 (11.5%)

**Table 3:** The total number and average number per patient of pain observations taken in the *standard group* and mean pain score recorded.

	Number of nurse observations	Mean number of pain observations per patient	Mean pain score (0–4)
Day 1 (*n* = 26)	253	9.73	0.57
Day 2 (*n* = 25)	352	14.08	0.14
Total 48 hours	605	10.25	0.32

**Table 4:** The total number and average number per day of pain observations taken in the *device group* and mean pain scores recorded on day 1 and day 2.

	Nurse observations	Device observations	Total	Mean nurse pain score (0–4)	Mean device pain score (0–4)	Mean total pain score (0–4)
Day 1 (*n* = 26)	269	118	387	0.41	1.47	0.73
Day 2 (*n* = 22)	319	183	502	0.14	0.48	0.26
Total	588	301	889	0.26	0.87	0.29

**Table 5:** Number of patients using each multimedia option and respective satisfaction score out of 10, with interquartile range, for each.

Use of multimedia components	Number using	Satisfaction
Information on pain and pain control	15	6 (5, 8)
Guided relaxation	13	6 (4, 8)
Games	13	5 (3.5, 6.5)
Guided stretch	12	7 (3.75, 8.25)
Talking book	10	4 (1.5, 6)
Music	10	5.5 (3, 7.75)
Film/TV	9	5 (1.5, 7)
Comedy	8	3.5 (0.25, 5)
Advice	4	1.5 (0, 4.5)

**Table 6:** Efficacy analysis: study feedback (information obtained from the 48-hour questionnaire).

Outcome	Group S	Group D
Worst reported pain since surgery; median (IQR) (scale 0–10)	8 (4.75, 10)	5.5 (3, 8)
Least reported pain since surgery; median (IQR) (scale 0–10)	1.5 (0.75, 3)	1.5 (0, 3.25)
Frequency of severe pain since surgery; median% (IQR)	25% (17.5%, 42.5%)	20% (7.5%, 42.5%)
Median (IQR) pain interference with^*∗*^		
Activities in bed	7 (2, 8)	5.5 (3, 7)
Breathing/coughing	4.5 (2, 8)	4 (1, 6.25)
Sleeping	4.5 (0.75, 6)	4 (1, 6)
Out of bed activities	5 (3, 7); *N* = 25	4 (1, 6); *N* = 25
Median (IQR) pain related^*∗∗*^		
Anxiety	5 (1.75, 7.25)	2 (0.75, 4.25)
Helplessness	5 (2, 7.25)	2.5 (0, 6.25)
Median (IQR) side effects^*∗∗∗*^		
Nausea	5 (1, 9.25)	2.5 (1, 7)
Drowsiness	8 (2.75, 9)	5 (1, 7.25)
Itching	0 (0, 3.25)	0 (0, 3.25)
Dizziness	4 (0, 8)	1.5 (0, 4.25)
Frequency of pain relief since surgery; median% (IQR)	80% (67.5%, 90%)	80% (70%, 90%)
Require more pain relief; *n* yes (%)	5 (19.2%)	7 (26.9%)
Information about pain treatment received; *n* yes (%)	19 (73.1%)	17 (65.4%)
Level of participation in pain treatment decisions; median (IQR)	8 (5.75, 10)	8 (3.25, 10)
Satisfied with the pain relief prescribed; median (IQR)	8 (7.75, 9.25)	8 (7.75, 10)
Satisfied with the pain control overall; median (IQR)	8.5 (7, 9.25)	9 (7, 10)

^*∗*^Score of 10 = completely interfered; ^*∗∗*^score of 10 = extreme; ^*∗∗∗*^score of 10 = severe.

## Additional Points

Postoperative pain is common and often difficult to manage. Taking a biopsychosocial perspective on pain suggests a role for nonpharmacological self-management methods as adjuncts. In a prospective, randomised control trial, elective surgical patients were given a handheld device, the Navimed, on which they could self-report pain and access software designed to help them manage their pain. Patients made full use of the Navimed to report pain and recorded higher scores on it than they reported to the nurses. Further work is required to extend these findings and to develop more sophisticated software for patient-identified needs.
