# Supplementary material for: Self-Managing Postoperative Pain with the Use of a Novel, Interactive Device: A Proof of Concept Study
Source: Pain Res Manag. 2016 Apr 30;2016:9704185. doi: 10.1155/2016/9704185 (PMC4904622; doi:10.1155/2016/9704185)
Supplement: Supplementary file 1 — The supplementary material provided is the post trial questionnaire given to all participants at the conclusion of their involvement. It asks questions regarding the retrospective experience of pain as well other symptoms and emotions using both closed questions and numerical rating scales. Those participants that used the Navimed were then asked a further series of questions specifically regarding their usage of, and satisfaction with the device. The results from this questionnaire are included within the main text of the article. [file 9704185.f1.pdf]

Appendix 1: Post Trial Questionnaire

Questionnaire - 48 hours

We would be grateful if you would participate in our survey on your experience of pain.

The aim of the survey is to improve the management of pain in this department.

Your participation is voluntary and the information you provide will be made anonymous once you hand in this questionnaire. This means that your name or other form of identification will be deleted from the questionnaire after you hand it in and will not be included in any records we will hold.

Your answers in this questionnaire will **not** be shared with your medical or nursing team.

Your team will treat you in the same way whether or not you choose to participate in our survey. If you decide not to complete the questionnaire it would be helpful to know the reasons (please use the space below).

Many thanks for taking part in this study. We would like to keep you informed about the progress of our study. If you would like this please write your email address or postal address below. (Your details will be held confidentially and used only in connection with this study and not available to anyone other than researchers in this study).

Email address:

Postal address:

Reason for not completing the questionnaire:

The following questions are about pain you experienced since your surgery.

P1. On this scale, please indicate the **worst pain** you had since your surgery:

0 1 2 3 4 5 6 7 8 9 10

no pain **worst pain possible**

P2. On this scale, please indicate the **least pain** you had since your surgery:

0 1 2 3 4 5 6 7 8 9 10

no pain **worst pain possible**

P3. How often were you in **severe pain** since your surgery?

Please circle your best estimate of the percentage of time you experienced **severe pain**:

0% 10% 20% 30% 40% 50% 60% 70% 80% 90% 100%

never in severe pain **always in severe pain**

P4. Circle the one number below that best describes how much, since your surgery, **pain interfered with or prevented you from**:

a. doing **activities in bed** such as turning, sitting up, changing position:

0 1 2 3 4 5 6 7 8 9 10

did not interfere **completely interfered**

b. **breathing deeply** or **coughing**:

0 1 2 3 4 5 6 7 8 9 10

did not interfere **completely interfered**

c. **sleeping**:

0 1 2 3 4 5 6 7 8 9 10

did not interfere **completely interfered**

d. Have you been **out of bed** since your surgery?

Yes No

If yes, how much did **pain interfere or prevent you from doing activities out of bed** such as walking, sitting in a chair, standing at the sink:

0 1 2 3 4 5 6 7 8 9 10

did not interfere **completely interfered**

Patient code:

P5. Pain can affect our mood and emotions.

On this scale, please circle the one number that best shows how much, since your surgery, **pain caused you to feel:**

a. **Anxious**

0 1 2 3 4 5 6 7 8 9 10

not at all **extremely**

b. **Helpless**

0 1 2 3 4 5 6 7 8 9 10

not at all **extremely**

P6. Have you had any of the following **side effects** since your surgery?

Please circle “0” if no; if yes, circle the one number that best shows the severity of each:

a. **Nausea**

0 1 2 3 4 5 6 7 8 9 10

none **severe**

b. **Drowsiness**

0 1 2 3 4 5 6 7 8 9 10

none **severe**

c. **Itching**

0 1 2 3 4 5 6 7 8 9 10

none **severe**

d. **Dizziness**

0 1 2 3 4 5 6 7 8 9 10

none **severe**

P7. Since your surgery, how much **pain relief** have you received?

Please circle the one percentage that best shows how much relief you have received from all of your **pain treatments** combined (medicine and non-medicine treatments):

0% 10% 20% 30% 40% 50% 60% 70% 80% 90% 100%

no relief **complete relief**

P8. Would you have liked **MORE pain treatment** than you received?

Yes No

P9. Did you receive any **information** about your **pain treatment** options?

Yes No

Patient code:

P10. Were you **allowed to participate in decisions** about your **pain treatment** as much as you wanted to?

0 1 2 3 4 5 6 7 8 9 10

not at all **very much so**

P11. Circle the one number that best shows how **satisfied** you are with the results of your **pain treatment** since your surgery:

0 1 2 3 4 5 6 7 8 9 10

extremely dissatisfied **extremely satisfied**

Pain control

1. Overall how would you rate your satisfaction with experience of pain control?

0 1 2 3 4 5 6 7 8 9 10

extremely dissatisfied **extremely satisfied**

2. Please can you write something in your own words on your experience of pain control on this admission.

Device

1. Did you have a device?

Yes No

If yes, please continue with the questions below.

If no, there are no further questions. Thank you for taking part.

2. Are you pleased that you had a device?

Yes No Don't know

3. How easy was the device to use?

very easy **easy** **neutral** **difficult** **very difficult**

4. Overall how would you rate your satisfaction with experience of the device?

0 1 2 3 4 5 6 7 8 9 10

extremely dissatisfied **extremely satisfied**

5. Did you use any **non-medicine methods** to relieve your **pain**?

Yes No

If yes, **tick all** that apply, and indicate how helpful you found it:

☐ **a. Information**

0 1 2 3 4 5 6 7 8 9 10

extremely unhelpful **extremely helpful**

☐ **b. Comedy**

0 1 2 3 4 5 6 7 8 9 10

extremely unhelpful **extremely helpful**

☐ **c. Guided stretch**

0 1 2 3 4 5 6 7 8 9 10

extremely unhelpful **extremely helpful**

☐ **d. Music**

0 1 2 3 4 5 6 7 8 9 10

extremely unhelpful **extremely helpful**

☐ **e. Film or TV**

0 1 2 3 4 5 6 7 8 9 10

extremely unhelpful **extremely helpful**

☐ **f. Talking book**

0 1 2 3 4 5 6 7 8 9 10

extremely unhelpful **extremely helpful**

☐ **g. Poetry**

0 1 2 3 4 5 6 7 8 9 10

extremely unhelpful **extremely helpful**

☐ **h. Guided relaxation/meditation**

0 1 2 3 4 5 6 7 8 9 10

extremely unhelpful **extremely helpful**

☐ **i. Games**

0 1 2 3 4 5 6 7 8 9 10

extremely unhelpful **extremely helpful**

5. Can you suggest any other non-medicine options that you may find helpful for pain?

6. Please could you write in your own words what effect(s) you think the device had (if it did)?

Thank you for your time and feedback
